# Longitudinal spatial dataset on travel times and distances by different travel modes in Helsinki Region

**DOI:** 10.1038/s41597-020-0413-y

**Published:** 2020-03-04

**Authors:** Henrikki Tenkanen, Tuuli Toivonen

**Affiliations:** 1 Digital Geography Lab, Department of Geosciences & Geography, University of Helsinki, Helsinki, Finland; 2 Department of Geography, University College London, London, United Kingdom; 3 Urbaria, Helsinki Institute of Sustainability Science, University of Helsinki, Helsinki, Finland

**Keywords:** Geography, Environmental economics

## Abstract

Comparable data on spatial accessibility by different travel modes are frequently needed to understand how city regions function. Here, we present a spatial dataset called the Helsinki Region Travel Time Matrix that has been calculated for 2013, 2015 and 2018. This longitudinal dataset contains travel time and distance information between all 250 metres statistical grid cell centroids in the Capital Region of Helsinki, Finland. The dataset is multimodal and multitemporal by nature: all typical transport modes (walking, cycling, public transport, and private car) are included and calculated separately for the morning rush hour and midday for an average working day. We followed a so-called door-to-door principle, making the information between travel modes comparable. The analyses were based primarily on open data sources, and all the tools that were used to produce the data are openly available. The matrices form a time-series that can reveal the accessibility conditions within the city and allow comparisons of the changes in accessibility in the region, which support spatial planning and decision-making.

## Abstract (summary)

**Table:** 

Measurement(s)	spatiotemporal_interval • Spatial Qualifier • travel time by public transport • travel time by private car • travel time by walking • travel time by cycling • travel distance by public transport • travel distance by private car • travel distance by walking • travel distance by cycling
Technology Type(s)	computational modeling technique • digital curation • directed network graph construction • node shortest path identification
Factor Type(s)	transport mode • year • time of day
Sample Characteristic - Environment	anthropogenic environment
Sample Characteristic - Location	Helsinki • Espoo • Vantaa • Municipality of Kauniainen

Machine-accessible metadata file describing the reported data: 10.6084/m9.figshare.11841462

## Background & Summary

Spatial mobility between locations is an inherent part of urban systems. Understanding travel times and travel distances between places is a key to analyse the city structure from a functional perspective. Travel times and distances also form the basis for measures of accessibility, which are often used as conceptual and methodological tools in spatial planning processes and decision making. Accessibility links e.g. landuse, transportation, socio-economic factors together in an easily understandable manner. Hence, accessibility assessment has become one of the “basic” analyses and part of the decision making process when planning new infrastructure or services in cities. Accessibility, which can be defined as “the potential for interaction”^1^, can be measured in various ways depending on the approach and the question at hand (e.g.^2–4^). However, time and metric distance are by far the most widely used measures of accessibility.

The ability to measure travel times and distances has evolved enormously during the past two decades. Simple Euclidian metrics have been replaced by advanced network-based measures^5^ that aim at modelling the distances and travel times for different modes of transportation^2^ as realistically as possible. Various advanced routing algorithms^6^ have been developed to help citizens to navigate through the cities, or to gain travel time information for different travel modes. However, using the same algorithms in broad-scale analyses requires high computational capacity due to the complexity of multimodal and multitemporal travel time estimation. Hence, planners and decision makers interested in general spatial patterns have often needed to rely on more simplistic distance measures^7^, such as atemporal Euclidian distances^8,9^, or considering only a single travel mode^10–12^.

Openness in data sharing has increased enourmously in the past decade or so^13^. Transportation data have been at the forefront of the openness and data are widely available from many parts of the world, including online data portals such as transitfeeds.com and transit.land, that provide public transport data from hundreds of cities across the world. These advances in data sharing has made it possible to develop sophisticated tools that support analysis of transportation and travel times in order to understand how dynamic cities function^8^. Increasing computational capacity (such as cloud computing and high performance computing) have made it possible to analyse data with higher accuracy and to a broader extent than ever before.

Stemming from these technical advancements and information needs, we introduce an open dataset called the Helsinki Region Travel Time Matrix, and a set of open source tools that were used to compute it. The dataset provides longitudinal spatial information for analysing accessibility in Helsinki Capital Region, comparably during 2013, 2015 and 2018. The Helsinki Capital Region (referred to as Helsinki Region in the rest of the manuscript) provides an interesting setting for transportation research, because many large-scale transportation infrastructure projects have been completed during the past five years, and the city region is growing rapidly. The data described provide windows to regional-scale accessibility realities with all common travel modes (public transport, car, bike, walking) in an easily-accessible format that can be linked with various other spatial datasets. The easy availability and usability of this information has led to the data being widely used by cities, services and businesses in the study area.

In this article, we have documented and shared the methodologies, tools and approaches that were used to produce the Helsinki Region Travel Time Matrix following the principles of open science. We provide detailed description of the dataset and its production, data quality assessment of the data against other data sources, and present examples of how the data can be used to provide insights into the urban dynamics and regional accessibility patterns. As the concept of accessibility naturally binds together questions of land use, transportation, economy and so on, the given dataset can deliver relevant information to support decision making in the study area.

## Methods

**Fig. 1:**
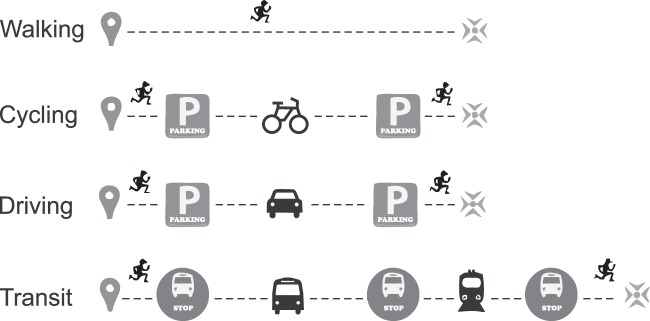
In door-to-door approach, every step of a journey in different travel modes is considered.

Being able to compare travel modes to each other (realistically) at different hours of the day requires following a so-called door-to-door approach (Fig. 1). This means that travel time and distance are calculated comparably between travel modes by considering every step of a journey when calculating a route between origins and destinations. These steps include walking legs and transfers from one vehicle to another and the time it takes to search for a parking space or lock/unlock one’s bike. Considering these steps makes the travel times between transport modes more comparable with each other, and realistic measures of accessibility can be provided.

**Fig. 2:**
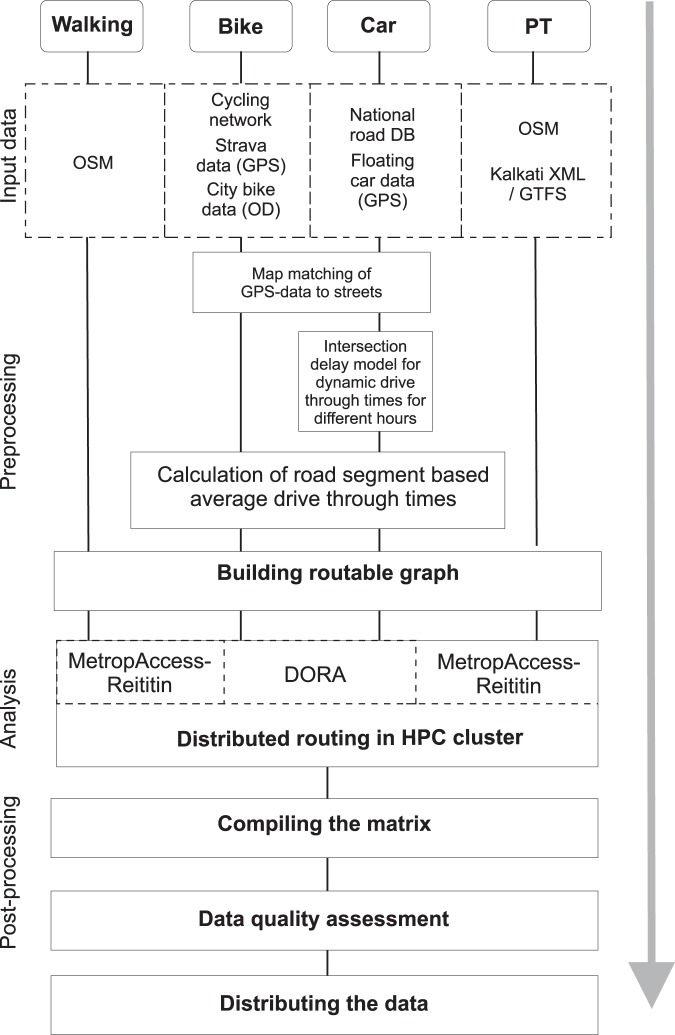
Generic workflow of the analytical steps that were taken to produce the travel time and distance data for Helsinki Region.

A variety of input datasets (Table 1) and methods were used to capture and model accessibility for different travel modes in a dynamic manner at different hours of the day. Figure 2 represents a generic workflow for producing the data. Specific preprocessing steps were applied for private car and cycling analyses to improve incorporation of the spatio-temporal dynamics of travel. After the initial pre-processing step, dedicated graphs were built for different travel modes that are required to calculate the shortest path routes between locations in the study area. Finally, the results were parsed and harmonized into a systematically formatted matrix, and the data quality was assessed against other data sources. The next sections provide further details about the analytical approaches used for each travel mode. Details on how to reproduce the data can be found at helsinkimatrix.github.io including processing codes and source data.

**Table 1:** Source datasets for the models.

Data	Description	Provider	Additional information
Kalkati XML	Public transport schedule data.	Helsinki Region Transport	developer.matka.fi/pages/en/kalkati.net-xml-database-dump.php
OpenStreetMap	Street network data used with public transport and walking.	OpenStreetMap	wiki.openstreetmap.org/
Digiroad	Street network data used with car calculations	Finnish Transport Infrastructure Agency	digiroad.fi
Strava cycling GPS data	GPS data from sport tracker application used in cycling analyses.	Strava	developers.strava.com/
City bike data	Origin-destination data from bicycle sharing system of city of Helsinki used in cycling analyses.	CityBike/City of Helsinki	www.citybikefinland.fi
Floating car data	Data were used to estimate the speeds and levels of congestion on different roads.	Helsinki Region Transport/City of Helsinki	https://www.hsl.fi/tutkimukset/liikenne-ja-matkustajalaskennat-ja-mittaukset
Origin-destination grid	Origin/destination locations based on 250 × 250 m statistical grid	Statistics Finland/Finnish Environment Institute	www.stat.fi/tup/ykraineistot/index.html

### Travel time calculations

**Fig. 3:**
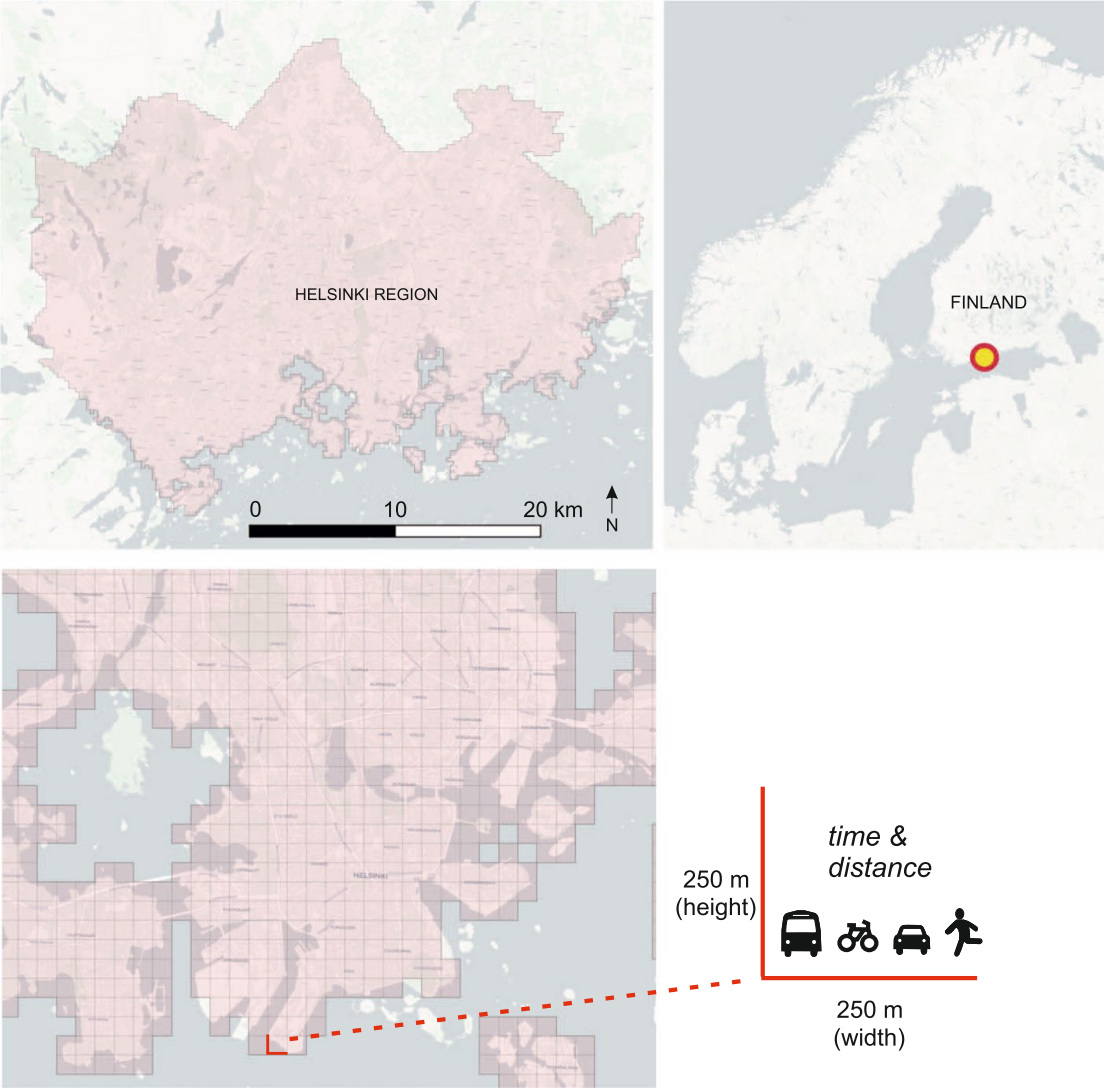
Dataset covers the Helsinki Region in Finland where the area has been divided into 250-metre statistical grid cells. Each grid cell contains information about the travel times and distances to every other cell in the area by public transport, car, bicycle and walking. Background map courtesy of OpenStreetMap contributors and Carto.

Travel time and distance calculations were done separately for each travel mode: public transport, private car, cycling and walking. Furthermore, the calculations were conducted separately for the morning rush hour (08:00–09:00) and midday (12:00–13:00) times, to be able to understand the dynamism of the transport system due to changes in timetables and congestion levels on the roads. The centroids of the statistical grid cells (Fig. 3) were used as origin and destination points for the calculations, covering the cities of Helsinki, Espoo, Vantaa and Kauniainen. The statistical grid (helsinkimatrix.github.io/grid) was originally created by Statistics Finland and the Finnish Environmental Institute (FEI), and it has the ETRS89/ETRS-TM35FIN projection. Hence, the data can be directly used with statistical data obtained from these institutions.

### Public transport

A dedicated tool called MetropAccess-Reititin (helsinkimatrix.github.io/reititin) was developed for public transport routings. As input data, MetropAccess-Reititin uses Kalkati.net XML data^14^ that is a specific data format used by Helsinki Region Transport for publishing public transport schedules, stops, routes etc. OpenStreetMap data were used for walking paths including areal features such as squares and plazas, and the data were obtained from Geofabrik^15^ in Protobuf format. MetropAccess-Reititin is written in JavaScript and it uses a modified Dijkstra’s algorithm that considers transit timetable information when finding the optimal public transport route between given origin and destination locations (see^16^ for details). MetropAccess-Reititin is openly available from helsinkimatrix.github.io/reititin.

The tool considers the entire travel chains (following the door-to-door approach) from the origin to the destination (see also Fig. 1):

- possible waiting at home before leaving
- walking from home to the transit stop
- waiting at the transit stop
- travel time to next transit stop
- transport mode change
- travel time to next transit stop
- walking to the destination

When routing, the tool first searches for the closest street edge from the origin (based on Euclidian distance). Following the network, it then finds 3–5 closest public transport stops, and considers all available transit options from those stops to find the fastest route between the given origin and destination points (for the given departure time). If the origin and destination are close to each other, the tool uses walking as the fastest route (whenever walking is faster than taking any of the public transport options). Walking speed was determined as 70 metres per minute. The tool stores detailed information about all steps of the journey that makes it possible to derive information about the exact distances for each travel mode. This makes it possible for example to calculate the CO_2_ emissions produced when using transit (see e.g.^17,18^).

For the travel time calculations, we obtained pareto optimal routes (due to varying departure times), by using ten departure times within the calculation hour using the so called Golomb ruler (departure minutes: 0, 1, 6, 10, 23, 26, 34, 41, 53, 55). The Golomb ruler makes it possible to gain maximal representation of departure times within one hour. The fastest route from these calculations is selected for the final travel time matrix. The travel times were calculated separately for the morning rush hour and midday on a typical work day using the effective schedules for those hours and days (see Table 2). Detailed documentation and instructions how to reproduce the public transport travel times/distances can be found at helsinkimatrix.github.io/pt_analyses.

**Table 2:** Dates and times that were used to calculate the public transport travel times/distance.

Year	Date	Hours
2018	Monday 29^th^ January	08:00–09:00 & 12:00–13:00
2015	Monday 28^th^ September	08:00–09:00 & 12:00–13:00
2013	Friday 8^th^ April	12:00–13:00 (only midday)

### Private car

For private car analyses, we first developed a dedicated intersection delay model (see^19^ for details) to generalise and associate the effect of congestion for the whole street network. In the model, we used GPS data from floating car measurements to understand how much congestion reduces driving speed at different parts of the study area (at different times). The deceleration effect of congestion is associated into the network by applying intersection penalties for different road classes and intersections. Hence, e.g. ramps at motorways have different intersection penalties compared to intersections affected by traffic lights on local main streets. By running the network through the intersection delay model, each road segment is given a different drive through times at different times of the day (rush hour, midday, whole day average, and speed limit based “freeflow” drive through times). The floating car measurements were collected by Helsinki Region Transport and the City of Helsinki. Preprocessed car network can be downloaded from helsinkimatrix.github.io/car_network.

When running the analyses with the modified street network, we followed the door-to-door approach, which includes:

- walking time from the real origin to the nearest network location (based on Euclidean distance)
- average walking time from the origin to the parking lot
- travel time from parking lot to destination
- average time for searching a parking lot
- walking time from parking lot to nearest network location of the destination
- walking time from network location to the real destination (based on Euclidean distance) See also Fig. 1.

For considering the time that it takes to walk to the car from home, we used values based on previous research conducted in Finnish city regions^20,21^. The distance was set as 180 metres (~2.5 minutes) in the city centre areas of Helsinki, and 130 metres in other areas (~2 minutes). The time that it takes to find a parking lot, was also based on previous literature^21^, and it was specified as 0.42 minutes across the study area. Calculations were done separately for two times of the day following rush hour (08:00–09:00) and midday (12:00–13:00) traffic conditions.

The calculations for 2018 were done with a dedicated open source tool called *Door-to-door Routing Analyst* (DORA) that was developed on top of PostGIS database v.2.3.3 (PostGIS community, 2019). DORA (helsinkimatrix.github.io/dora) is an open source multimodal routing tool that uses door-to-door approach when retrieving travel times between multiple origins and destinations. It can be used to route car, cycling and walking routes and is able to read any road network setup in a database with the pgRouting v2.3.2 (pgRouting community, 2019) extension. Detailed documentation for reproducing the data can be found at helsinkimatrix.github.io/car_analyses. Car routing for 2013 and 2015 was conducted with the ArcGIS v.10.1 (Environment Systems Research Institute, 2012) Network Analyst tool using a dedicated toolbox developed for the purpose. The ArcGIS toolbox (requires ArcGIS -software) is openly available at helsinkimatrix.github.io/md-tool. We ensured that the DORA and ArcGIS routing tools produced similar results, making the datasets between the years comparable; see helsinkimatrix.github.io/dora_validation for further details.

### Cycling

For cycling analyses, we built a customized routing network called *MetropAccess-CyclingNetwork* by utilising GPS data from the Strava sport tracker application from 2016. The Strava dataset is based on data from 5223 unique users. We first linked the GPS points to the closest streets by using customised map matching techniques^22,23^. We then identified the roads that are most commonly used by cyclists and calculated user-based cycling speeds for different road segments. Finally, we aggregated this information into an average travel speed per segment. See^22,23^ for details on how the Strava data was processed.

Since the cycling speed is heavily influenced by personal characteristics of the cyclist (e.g. gender, fitness, age, etc.), the cycling speed we identified was not used directly. Instead, we associated each road segment with information about how much faster or slower the cyclists typically ride at a given road segment compared to the total average travel speed across the network according the Strava data. For instance, segment A is ridden 10 percent faster than on average, and along segment B the speeds are typically 5 percent slower than the baseline average speed.

Once the speed profiles were linked to road segments, we calculated ride through times by using the average cycling speed (19 km/h) of Strava sport tracker users as the reference value for “fast cyclist”. We also calculated separate ride through times for “slow cyclist”, in which we used the average cycling speed of a city bike system users (12 km/h). The average travel speed for city cyclists is based on data from a highly popular bicycle sharing system (BSS) in Helsinki. The data contains information about the origin and destination bike station, as well as distance travelled and the time it took. This information was used to calculate the typical travel speed of city cyclists. We also included extra time (1 minute) for unlocking and locking the bike, in order to follow the door-to-door principle. The unlocking/locking time is a naïve measure, as it is the same for every location. However, because of this, it is easy to modify the time if needed, or if more accurate information is available.

Due to privacy reasons and our licensing agreement with Strava, we cannot publicly share the raw Strava data. However, the preprocessed cycling network that contains segment wise travel speeds based on Strava data can be downloaded from helsinkimatrix.github.io/bike_network. The Python scripts that were used to produce the cycling network can be found at helsinkimatrix.github.io/bike_preprocess. Documentation for reproducing the cycling travel times and distances with DORA routing tool is available at helsinkimatrix.github.io/bike_analyses.

### Walking

The walking routes were calculated using the MetropAccess-Reititin by disabling all motorised transport modes in the calculation. Thus, all routes are based on the OpenStreetMap (OSM) network obtained from^15^ at the time of the analysis. OSM is a good data source for estimating pedestrian travel times especially in urban areas^24^, as the data contain paths that are not available in the national road network (which is specifically targeted at car drivers). We used a static walking speed of 70 metres per minute (4.2 kilometres per hour) that is also used by public transport journey planner in Helsinki Region (reittiopas.hsl.fi). The same walking speed was applied also for the walking legs in the public transport calculations. Further details on how to reproduce the data for walking can be found at helsinkimatrix.github.io/walk_analyses.

## Data Records

**Fig. 4:**
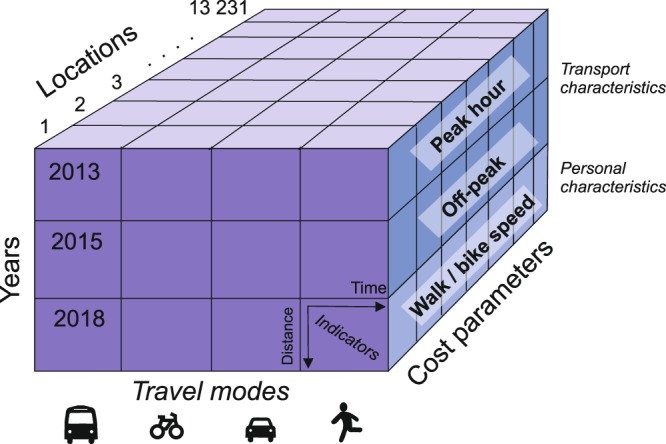
A data cube representation shows the different dimensions of the described dataset.

The Helsinki Region travel time matrix is a multidimensional dataset that provides information about accessibility from multiple angles. Figure 4 illustrates these dimensions using a data cube representation. The dataset for each year is fundamentally a 13231-ordered tensor covering accessibility matrices for 13 231 statistical grid cells for 2013, 2015 and 2018. The data^25^ can be download from Zenodo, an open data repository maintained by CERN.

Each matrix contains accessibility information (travel times and distances) from every grid cell in the region to the given target cell by different travel modes (public transport, bike, car, and walking). To consider diurnal dynamics, the indicators have been calculated separately for the morning rush hour and midday times for public transport and private car. The data contains 175 059 361 rows and 18 columns (see Table 3). Hence, the data contains approximately 3.15 billion individual values for year 2018. The number of values is lower for 2015 and 2013 due to lower dimensions: the 2015 dataset does not contain cycling information; the 2013 dataset does not contain cycling information, nor accessibility information for the morning rush hour.

**Table 3:** Attributes of the dataset.

Column	Description
from_id	ID number of the origin grid cell
to_id	ID number of the destination grid cell
walk_t	Travel time in minutes from origin to destination by walking
walk_d	Distance in metres of the walking route
bike_f_t	Total travel time in minutes from origin to destination by fast cycling; Includes extra time (1 min) that it takes to take/return bike
bike_s_t	Total travel time in minutes from origin to destination by slow cycling; Includes extra time (1 min) that it takes to take/return bike
bike_d	Distance in metres of the cycling route
pt_r_tt	Travel time in minutes from origin to destination by public transportation in rush hour traffic; whole travel chain has been taken into acount including the waiting time at home
pt_r_t	Travel time in minutes from origin to destination by public transportation in rush hour traffic; whole travel chain has been taken into account excluding the waiting time at home
pt_r_d	Distance in metres of the public transportation route in rush hour traffic
pt_m_tt	Travel time in minutes from origin to destination by public transportation in midday traffic; whole travel chain has been taken into acount including the waiting time at home
pt_m_t	Travel time in minutes from origin to destination by public transportation in midday traffic; whole travel chain has been taken into account excluding the waiting time at home
pt_m_d	Distance in metres of the public transportation route in midday traffic
car_r_t	Travel time in minutes from origin to destination by private car in rush hour traffic; the whole travel chain has been taken into account
car_r_d	Distance in metres of the private car route in rush hour traffic
car_m_t	Travel time in minutes from origin to destination by private car in midday traffic; the whole travel chain has been taken into account
car_m_d	Distance in metres of the private car route in midday traffic
car_sl_t	Travel time from origin to destination by private car following speed limits without any additional impedances; the whole travel chain has been taken into account

The datasets are distributed separately for each year as compressed zipfiles. The data have been divided into 13231 text files according to the destinations of the routes. The datafiles have been organized into subfolders that contain multiple (approx. 4–150) matrix files. Individual folders consist of all the travel time matrices that have same first four digits in their filename (e.g. 5785xxx). The dataset is licensed with the Creative Commons 4.0 BY license that allows its use for any purpose (including commercial use) as long as appropriate credit for the dataset is stated.

**Fig. 5:**
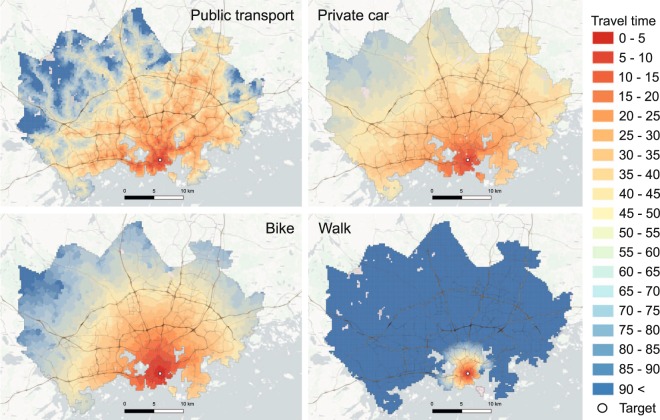
Comparable travel time surfaces for a given target (marked with a point symbol) reveal the areal travel time differences between travel modes. Background map courtesy of OpenStreetMap contributors and Carto.

Table 3 represents all attributes of the dataset and their description. Accessibility information can easily be linked back to the statistical grid for visualisation purposes based on the *from_id* column, which contains a unique id for each statistical grid cell in the area. Each matrix layer contains travel time (and distance) information to a single target. Data are distributed as separate text files for each target grid cell that are named after the target grid cell-id (i.e. *to_id)*, such as *travel-times-to-5975375.txt*. In the text file, the separator is semicolon (‘;’), and no-data is reported with value −1. Visualising this information on a map reveals the regional accessibility patterns in the Helsinki Region by different travel modes, and makes it easy to compare the travel modes to each other (Fig. 5). The statistical grid can be downloaded from helsinkimatrix.github.io/grid for visualisation purposes.

Table 4 represents basic statistical characteristics of the data. Because we did the calculations from every grid cell to every other grid cell in the study area, it is possible to extract the fundamental performance characteristics of the whole transportation system in the region. These statistics reveal that the average travel time between all statistical grid squares in the Helsinki Region during rush hour (2018) was 84.3 minutes by public transport and 44.5 minutes by private car. Comparing these values to average cycling travel time by fast cyclist (59.1 minutes) reveal that cycling is a highly-competitive travel mode: it is faster than public transport on average, and only 15 minutes slower than car during rush hour. However, these travel times assume that the cyclist would be able to maintain the fast average cycling speed of 19 km/h.

**Table 4:** Statistical characteristics of the transportation system in Helsinki Region for different transport modes and years.

Travel mode	Mean t	Median t	Std t
***Public transport***			
*Rush hour*			
*2018*	84.3 minutes	81 minutes	34.4 minutes
*2015*	82.9 minutes	79 minutes	34.2 minutes
*2013*	—	—	—
*Midday*			
*2018*	86.2 minutes	83 minutes	34.4 minutes
*2015*	84.3 minutes	81 minutes	33.6 minutes
*2013*	81.3 minutes	78 minutes	32.4 minutes
***Private car***			
*Rush hour*			
*2018*	44.5 minutes	44 minutes	16.5 minutes
*2015*	41.9 minutes	41 minutes	14.2 minutes
*2013*	—	—	—
*Midday*			
*2018*	38.9 minutes	38 minutes	14.3 minutes
*2015*	37.2 minutes	36 minutes	12.5 minutes
*2013*	37.7 minutes	37 minutes	12.6 minutes
*Free flow*			
*2018*	25.6 minutes	25 minutes	8.9 minutes
***Cycling 2018***			
*Fast biker*	59.1 minutes	57 minutes	27.6 minutes
*Slow biker*	93.2 minutes	89 minutes	43.6 minutes
*Walking*			
*2018*	281.9 minutes	271 minutes	133.2 minutes
*2015*	282.0 minutes	271 minutes	133.3 minutes
*2013*	281.0 minutes	269 minutes	135.6 minutes

As we calculated the accessibility information for three years, following the same methods and similar datasets, it is possible to identify overall trend in the performance of the transportation system in the area. The statistics show that the travel times in the study area have been increasing slightly during the five-year time-period (2013–2018). Average travel time by public transport at midday has increased from 81.3 minutes to 86.2 minutes, whereas the travel time by private car (midday) has increased from 37.7 to 38.9 minutes. The changes are modest, but they can reveal how the changes in the transportation infrastructure affects the overall performance of the system from the travel time perspective.

## Technical Validation

**Fig. 6:**
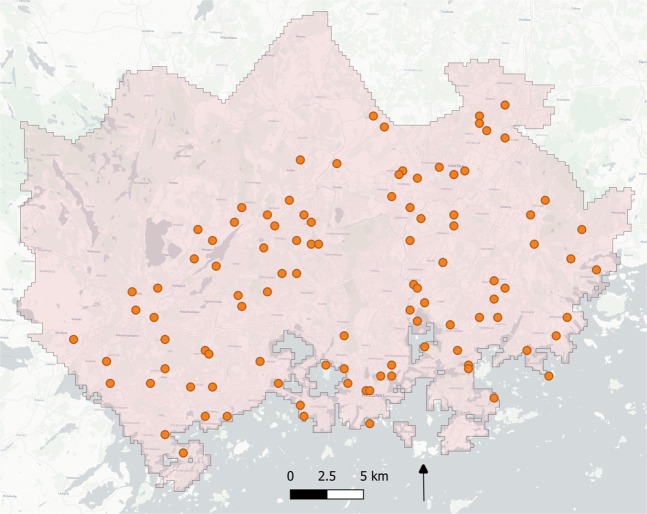
Validation points (n = 100) in the region that were chosen randomly. Trips between all these locations were assessed. Background map courtesy of OpenStreetMap contributors and Carto.

We validated the data quality systematically by comparing the travel time estimates to other data sources (see helsinkimatrix.github.io/validation) based on a random sample of 100 locations in the region (Fig. 6). To estimate the data quality of our public transport travel time estimates, we calculated routes between all sample points (9 900 origin-destination connections) using MetropAccess-Reititin and OpenTripPlanner v1.3 (OpenTripPlanner community, 2019). We used identical schedules with both tools, i.e. 29 January 2018 at 7 am with 10 departure times according the Golomb ruler (see Methods section). OpenTripPlanner is a widely used public transport journey planning tool that is also used by Helsinki Region Transport.

For private car, we compared our rush hour car travel times to drive times obtained from the Google Maps website (www.google.com/maps). Travel time estimates from Google Maps take into account traffic conditions on the roads at different times of the day and provides typical travel times for given hour of the day. Travel times on Google Maps are reported as time ranges (such as, “typical travel time is 22–28 minutes”) where the lower value represents time without congestion effect, and the upper value represents travel time in which congestion has affected the trip duration. We calculated the arithmetic mean travel time based on the lower and upper values of Google’s travel time estimates following the traffic conditions of Monday 6 May 2019 (a normal workday). Furthermore, we added an additional 3 minutes to these travel times to approximate the time it takes to find a parking space and walk to/from the car (following the door-to-door approach). We then compared these values against average travel time estimates based on the DORA tool using the same approach. We calculated the average travel times as an arithmetic mean based on the freeflow (column *car_sl_t*) and rush hour travel times (column *car_r_t*).

For cycling and walking, we compared our travel time estimates to OpenTripPlanner using the same walking (70 metres per minute) and cycling speeds (19 km/h) that we used to produce the dataset. Because our cycling travel time estimates are based on a custom cycling network which is adjusted according to the observed travel speeds of Strava users, we also validated the cycling using OpenStreetMap data without any customisation. In this way, could evaluate if there are differences in the travel time estimates due to algorithmic differences/routing tools. For cycling, an additional minute was added to OpenTripPlanner time estimates to approximate the time it takes to lock/unlock a bicycle, following door-to-door approach.

**Fig. 7:**
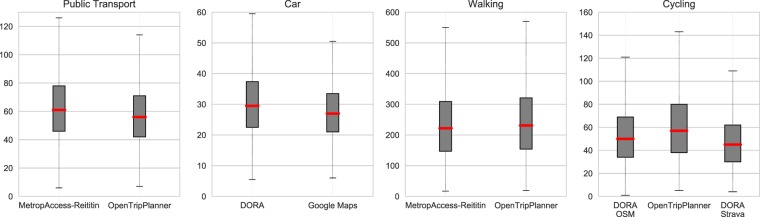
Boxplots showing the distribution of overall travel times based on validations against OpenTripPlanner and Google Maps. The results show that the travel time estimates produced by different routing tools, produce similar results as the first and third quartiles in the comparisons are quite similar. Also the means (red line) are close to each other having less than 10% difference with all travel modes except cycling.

Figure 7 shows that, in general, MetropAccess-Reititin produces slightly higher (6 percent) travel times by public transport with average travel time of 62.2 minutes compared to OpenTripPlanner’s 58.3 minutes mean travel time. Car travel time estimates based on DORA are on average three minutes higher (10 percent) compared to estimates obtained from Google Maps. Walking travel time estimates based on MetropAccess-Reititin are on average 9 minutes shorter (4%) than the travel times calculated by OpenTripPlanner. Cycling travel times based on DORA are on average 7.6 minutes shorter (13%) compared to OpenTripPlanner estimates when using OpenStreetMap data (without any modifications), and 13 minutes shorter (22%) when using customised cycling network based on Strava data. These values are based on the overall travel times of the random sample, and do not consider trip-wise differences.

**Fig. 8:**
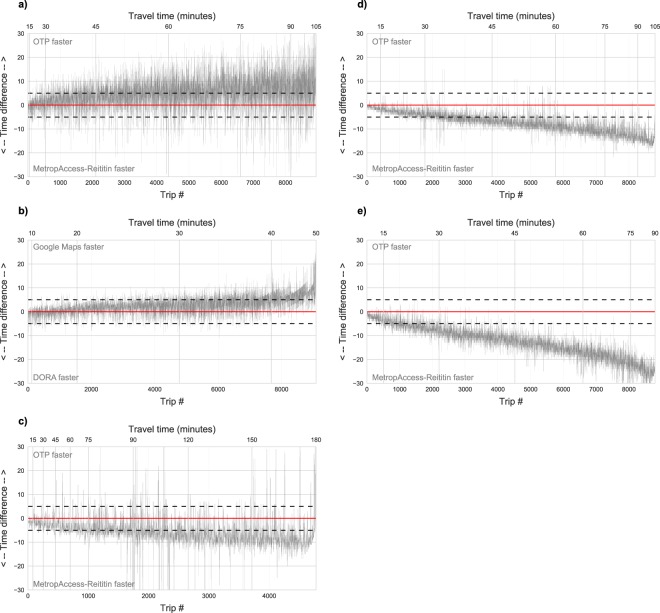
Systematic travel time comparisons were conducted with MetropAccess-Reititin, OpenTripPlanner, DORA and Google Maps to evaluate the data accuracy. The plot shows travel time differences in minutes trip by trip between randomized validation points by public transport (**a**), private car (**b**), walking (**c**), cycling with OSM network (**d**) and cycling with Strava network (**e**). The differences between the routing tools’ time estimates are within ±5 minutes if the observation is located between the dashed black lines. With car and public transport, MetropAccess-Reititin and DORA tend to produce slightly higher travel estimates on longer trips, while with walking and cycling they tend to produce lower travel times.

Figure 8 represents the differences between the tools in more detail by showing trip-wise differences when the travel times of each trip between origins and destinations are compared to each other. Trips are ordered by the duration of the trip (see the upper x-axis labels) to assess if the similarity in travel times changes depending on the length of the trip. Results show that MetropAccess-Reititin and DORA produce results similar to those produced by OpenTripPlanner (OTP) and Google Maps with all travel modes up to 30 minutes travel time from origin (within ±5 minutes range). On longer trips, the difference (and variance) in travel times increases, and the travel time estimates in the matrix tend to be slightly higher with car and public transport (Fig. 8a,b), although this fluctuates trip by trip. However, the travel times tend to be slightly lower in the matrix by walking and cycling (Fig. 8c,d). The reason for travel time differences after 30 minutes is most probably due to algorithmic differences to find the most optimal route: OTP uses the A* routing algorithm, whereas MetropAccess-Reititin and DORA use Dijkstra’s algorithm.

In addition, differences between the datasets used for routing causes differences. In terms of car analyses, Google uses its own street network, which differs from OpenStreetMap and Digiroad, and they also utilise real-time and historical GPS data from mobile phones to produce the travel time estimates, whereas our car travel time estimates are based on a generalized model that has been built on top of floating car measurements. There are also clear differences between travel time estimates for cyclists. This can be seen from the faster cycling travel times with the Strava-adjusted street network that tend to produce faster travel times than the OSM street network which has not been modified in any way, and the travel times are based on static travel speed and length of the road segments (Fig. 8e). OTP tend to produce higher travel times than DORA when using an unmodified OpenStreetMap dataset (Fig. 8d). We believe this difference is related to how the routing network is generated by default for cycling in OpenTripPlanner. OTP is more selective in terms of the roads that will be used when routing with bike as there are specific weights applied to roads to produce more “bike friendly” results. In our data, this approach has not been used and the results represents the unweighted shortest path time estimates. Further details about bike routing in OTP can be found from https://wiki.openstreetmap.org/wiki/OpenTripPlanner#Basic_Permissions.

### Considerations on data quality

When assessing the differences between different data sources and tools (see Validation), it is important to understand that there is no absolute and “correct” travel time between locations, as the routes and their conditions are always generalisations of reality. Although current data sources and algorithms can provide accessibility information more accurately than ever, it is important to note, that pure chance (or “bad luck”) might also have a significant role in increasing (or sometimes decreasing) significantly the travel time from one location to another. In addition, it is important to understand that travel times by walking and cycling are greatly influenced by personal characteristics, such as age, fitness, gender and how experienced a cyclist the person is^22,26^.

One limitation in the dataset is that there are obvious gaps in the data considering a range of dimensions. If one’s interest would be to make longitudinal comparisons e.g. between years 2013 and 2018, the current dataset provides limited number of dimensions for these analyses, namely including midday travel times and distances by public transport, car and walking. Hence, cycling information is only available for 2018, and rush hour indicators are only available for 2015 and 2018.

The comparability of the indicators between travel modes relates to the quality of the available input data. Data used to estimate the congestion levels, or the time that it takes to find a parking space in different parts of the city, or how much time it takes to get and unlock a bike when departing from home, all affect the results. Furthermore, obtaining good quality data about these factors can be difficult, which might be a challenge in terms of producing a similar dataset elsewhere.

## Usage Notes

While our dataset covers the Helsinki Region in Finland, the workflow presented is generally applicable to generating similar datasets in other regions. Globally-available transit schedule datasets following standards such as General Transit Feed Specification (GTFS)^27^ or Network Timetable Exchange (NeTEx)^28^, OpenStreetMap^29^ road network data, and multimodal routing tools such as OpenTripPlanner make it possible to conduct similar analyses in thousands of cities across the world. For cycling, Strava’s API^30^ and heatmap^31^ serve to retrieve relevant information about cycling speeds in various parts of the world. Openly available traffic data such as Uber Movement^32^ can be used to retrieve information about traffic conditions and car travel speeds in different parts of the world.

The dataset presented here allows a variety of different kind of analyses of accessibility. When combined with other data sources, such as demographic data, it is possible to extract a variety of policy relevant information about the characteristics and developments of the city region.

### Spatial differences in accessibility patterns

**Fig. 9:**
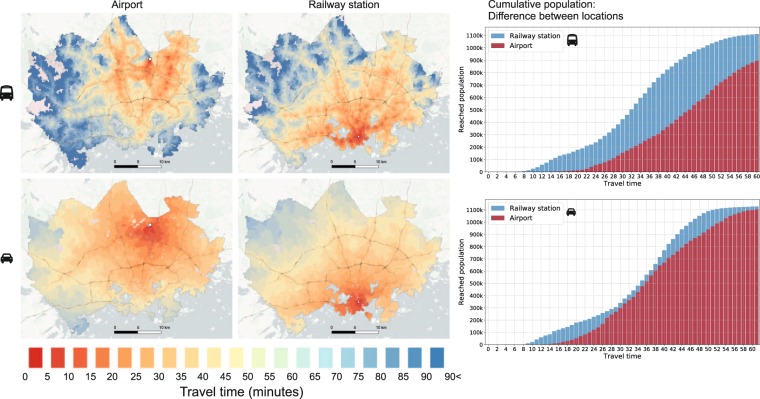
The dataset can be used to reveal how accessibility patterns differ at different locations. These maps demonstrate how the travel times to airport and railway station differs with public transport and car. Background map courtesy of OpenStreetMap contributors and Carto.

One of the most obvious use cases for the data is to investigate how accessible locations are. This allows questions like, is location A more accessible than B by travel mode X to be answered. The maps in Fig. 9 represents the travel times to two locations in the study area (airport and central railway station). The patterns reveal clearly how the accessibility patterns vary between locations. As the calculations were based on centroids of the statistical grid, it is easy to link accessibility information e.g. with population data. The cumulative population graphs in the Fig. 9 reveal the number of individuals reaching the locations within given travel time, and makes it possible to detect differences between locations.

### Detecting changes with time series data

**Fig. 10:**
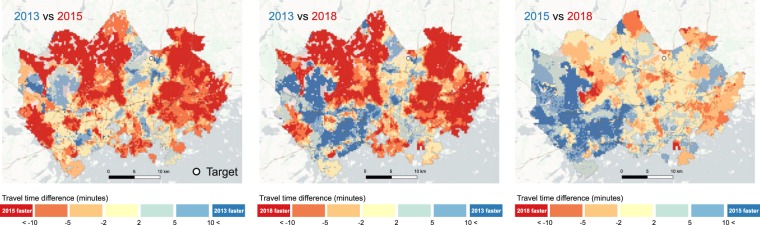
Annual changes in accessibility to the Helsinki-Vantaa airport reveal significant improvements in travel time between 2013 and 2015. The new metro line that was to the west of the region and is present in the data for 2018 reveals the increased travel times in 2018. Background map courtesy of OpenStreetMap contributors and Carto.

The Helsinki Region Travel Time Matrix has been produced for three years: 2013, 2015 and 2018. As the datasets follow the same methodological approaches, it is possible to detect changes in the transportat system that have occured in the region during the five-year period. Figure 10 represents an example of the annual differences in accessibility for the selected destination (Helsinki-Vantaa airport) using midday travel times. The maps reveal clear improvements of accessibility to the airport in most of the region (due to a new direct rail connection), but also a decline in the levels of accessibility in the western part of the region. The decline occured after a new metro line to the west of the region opened in 2017. This generally increased the travel times to the airport as more people needed to make transfers between busses and the metro. In a similar manner, the dataset would allow investigation of diurnal changes (comparing rush hour times to midday times) and detecting temporal changes by different travel modes.

### Travel time differences between travel modes

**Fig. 11:**
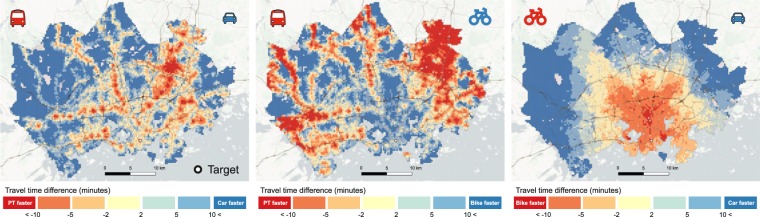
Travel time comparisons between travel modes reveal the areas where a given transport mode is faster than the other. Background map courtesy of OpenStreetMap contributors and Carto.

The door-to-door approach makes it possible to compare travel modes realistically. Figure 11 represents three maps comparing public transport, private car and cycling to each other. The maps reveal that there are clear differences between travel modes, but the regional patterns vary depending on which modes are compared.

### Detecting the most central areas by travel mode

**Fig. 12:**
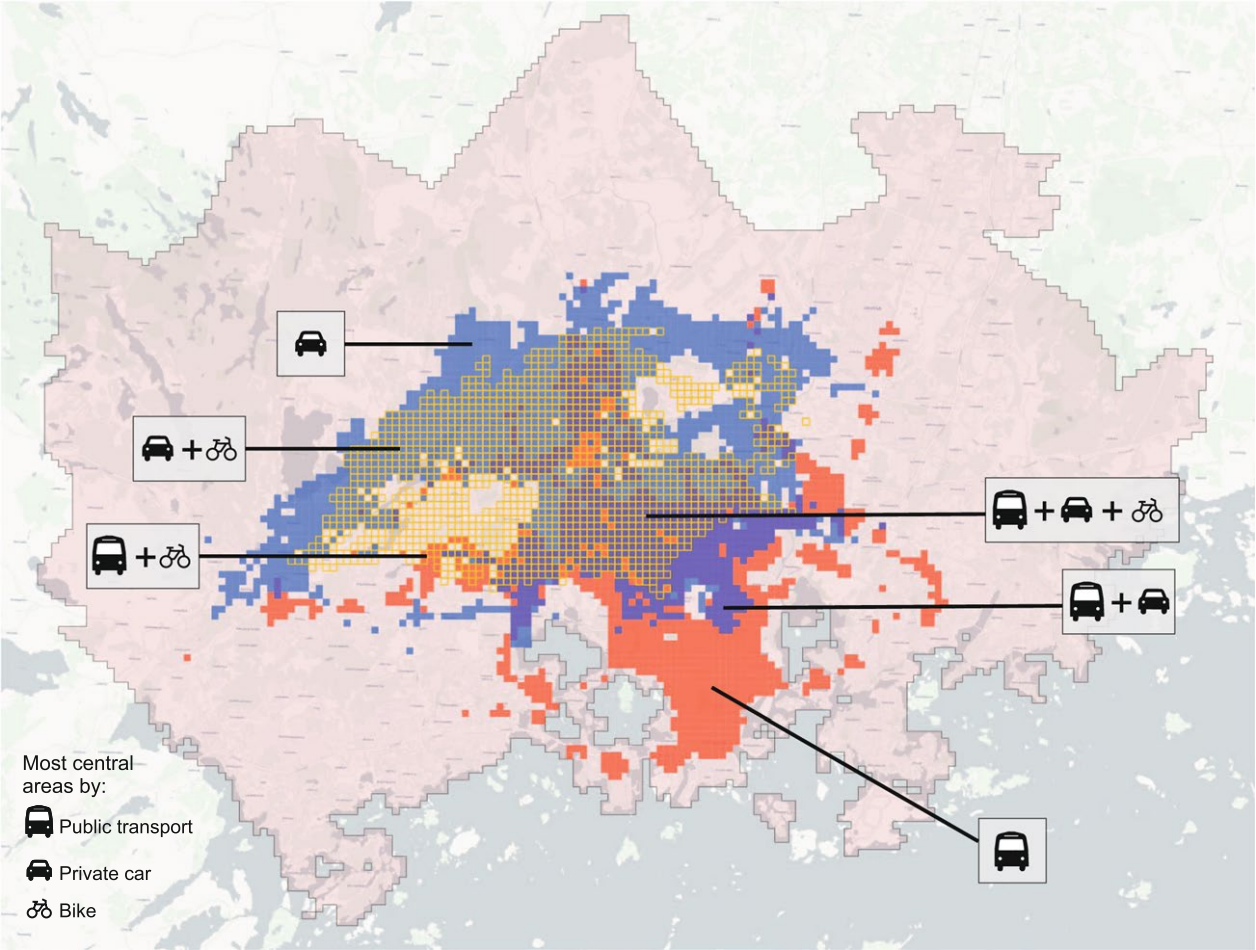
The most accessible 10 percent of the grid cells in the area by different travel modes (as median travel time). Close to the topological centre of the region, most of the areas overlap each other. Background map courtesy of OpenStreetMap contributors and Carto.

The Helsinki Region Travel Time Matrix is fundamentally a 13231-dimensional matrix from the spatial perspective. This makes it possible to extract rich system-level information from the data considering different transport modes (such as the statistics in Table 4). Figure 12 presents the most accessible areas (best 10 percent) in the study region by public transport, car and walking (including overlapping areas). The centrality has been calculated with simple map algebra using local operation through the 13231-dimensional data. As an output, median travel time was calculated for each location, and the best 10 percent of those values were selected for this map. The map reveals that the most central areas in the study area vary significantly depending on the transport mode. The most central areas by public transport are located more to the south and extend to a few spots with good rail connections, whereas the most central areas by car are concentrated more to the north where the ring roads and major highways are located. The most central areas by cycling follow the natural centrality of the street network in the given study area quite closely. This is an important finding e.g. for city planners and traffic planners when thinking about the equity of service access or focus areas for public transport development.

### Location-allocation

**Fig. 13:**
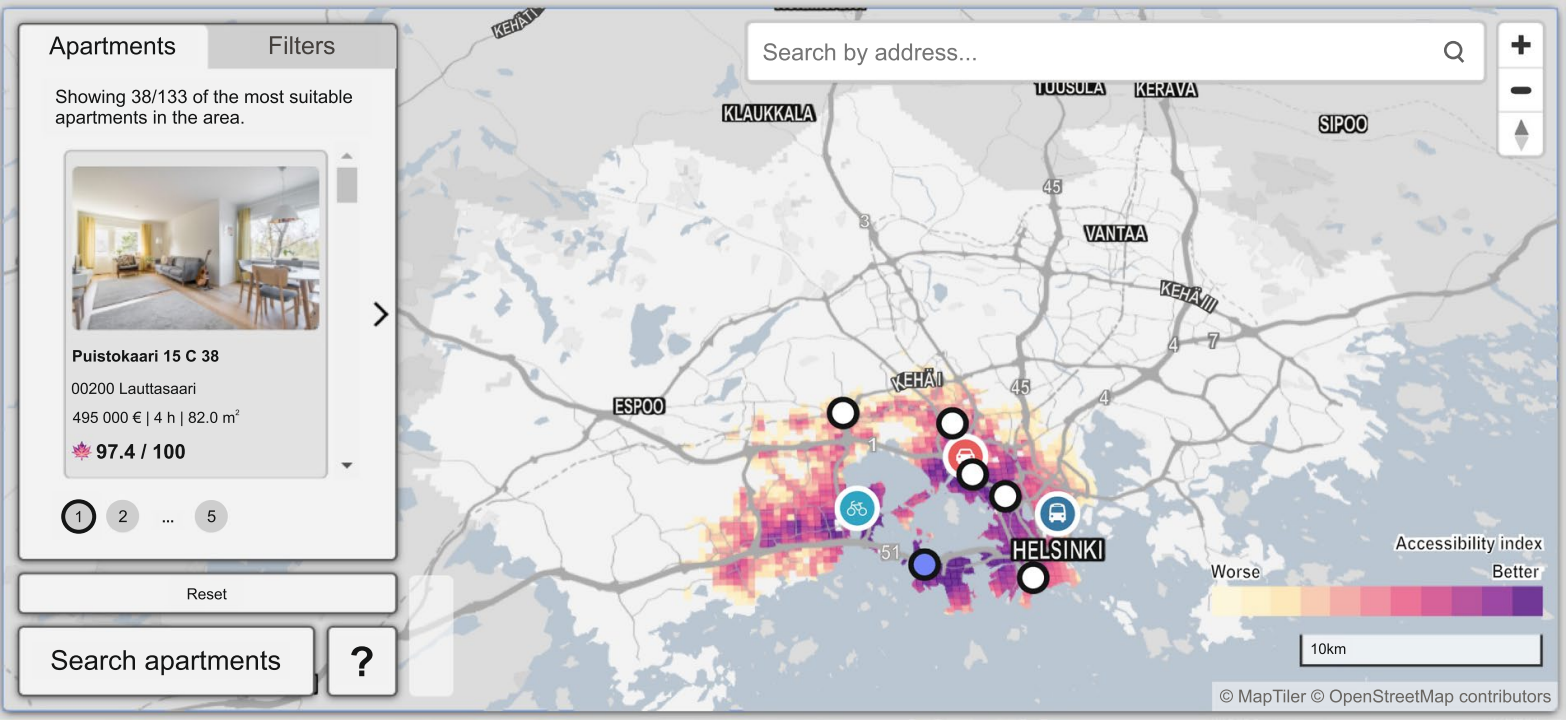
A snapshot from an “Apartment search engine” developed by Mapple Analytics Ltd that utilizes multidimensional accessibility data to make recommendations of the most suitable apartments by minimizing the time spent with commuting. Background map courtesy of OpenStreetMap contributors and Carto.

Accessibility is often one of the key criteria for finding an optimal location for a specific purpose. The dataset described here makes it possible to conduct sophisticated location allocation analysis based on multiple criteria. Figure 13 shows an example of such analysis, in which accessibility information combining different travel modes have been used together to find the most suitable apartments in the Helsinki Region. In this example, we integrated the accessibility information in a search tool that identifies optimal locations by minimizing the need for travel to commonly-visited places such as work, hobbies, school etc. Users can select the points of interest from the map as well as the travel mode they typically use to travel to the given location and the number of times the location is visited per week. As a result, the tool finds the most suitable areas in which to buy an apartment and ranks the available apartments based on this area.

## Data Availability

All the tools and source codes for the accessibility tools used for producing these datasets are openly available from the following links: MetropAccess-Reititin: helsinkimatrix.github.io/reititin DORA: helsinkimatrix.github.io/dora In addition, the full analytical workflow that was used to produce these matrices are published openly that makes it possible for anyone to assess the process pipeline: helsinkimatrix.github.io
